# Treatment of Fracture of the Jaw, with Critical Remarks

**Published:** 1880-09

**Authors:** Thomas Brian Gunning

**Affiliations:** New York


ARTICLE VI. PART IV.

### TREATMENT OF FRACTURE OF THE JAW, WITH CRITICAL REMARKS.

BY THOMAS BRIAN GUNNING, D. D. S., NEW YORK.

This was given by Malgaigne to show the method by which the displaced condyle may be reduced and held in place; it has, however, been referred to as a means of diagnosis, and I therefore take it into consideration in this connection, especially as the sooner such nonsense is eliminated from our text books the better both for practitioners and patients.

In the first place Dr. Packard's translation puts this case before the reader in quite a different aspect to that presented by Professor Hamilton, who omits the last paragraph, in which Malgaigne says:

" Ribes, to whom this plan is due, had occasion to apply it in the case of a cannonier who had a fracture of the neck of the right condyle with a wound of the corresponding cheek, and another fracture on the left side of the body of the jaw. The destruction of the soft parts gave him the idea of carrying the finger to the inner side of the condyle in order to replace it, which he did easily; and the patient recovered without deformity at that portion of the bone."*

Now the true sense of Malgaigne's report is that by the destruction of the soft parts a way was open for the finger, which in the ordinary state of the cheek does not exist.

But by the omission of this part of his report, Professor Hamilton's readers are led to suppose that the condyle can be reached and set from the inside of the mouth in the usual condition of the parts, and it must be shown why it cannot.

Before doing this it is necessary to correct the erroneous statements in respect to the condyle remaining still in the glenoid cavity while the pterygoid muscle makes it rotate and carry its fractured neck upward and forward. It has been shown that in health the condyle with its crowning interarticular fibro-cartilage moves forward to open the mouth, while the back border of the ramus opposite the lobule of the ear remains still, and that the lower part of the ramus passes back during the descent of the front of the jaw.

* Packard's Edition of Malgaigne Vol. I. p. 325.

Further, the case recently spoken of, in which part of the ramus was lost through necrosis following gun shot wound, shows the same effect of the muscular action, the condyle making the forward movement, or turning upon a point below in the spine of the back border of the ramus. It follows that if, after fracture of the neck of the condyle, the inferior fragment is in contact with the back of the condyle, it is owing either to the displacement at the time of fracture or the direction of the fractured surfaces, or both, and this displacement maintained by the temporal, masseter and internal pterygoid muscles, etc., drawing the inferior fragment up and back; *not*, however, by the action of the external pterygoid bringing the condyle and its neck into a horizontal position, as stated.

It has already been shown that fracture in the ramus cannot be explored in the mouth beyond the ascending curve of the internal oblique ridge. This of course does not prevent the entrance of the finger beyond the fauces, it being well known that its point can reach the back of the pharynx and also into the extreme upper back corners; but the back wall of the adult pharynx is less than two inches across from side to side, while the styloid process of the temporal bone is about twice this distance from its fellow on the opposite side, and each one about half an inch *back* of the condyle. This is so far, that the finger could hardly reach the styloid process even if it were not covered in by a mass of muscles and other tissues, including the internal caroted artery, and internal jugular vein, with important nerves, etc.

In brief, the directions of both Malgaigne and Hamilton for finding the styloid process are all erroneous and impracticable.

We now pass to consider the feasibility of reaching the condyle with its fractured neck turned upward and forward as stated in the directions now under review. When the finger is carried along the gum on the inside of the upper third molar, if its point is pressed into the commencement of the soft palate, it will feel the hamular process on the lower end of the *internal pterygoid plate* of the sphenoid bone, this hamular process being distant from its fellow on the opposite side about one inch and a half.

Now the ramus just behind and above this process is naturally a full inch outside of it, and if the neck of the condyle, or any part lower down, were fractured, but not displaced, no decision could be arrived at from examination inside the mouth; and if the condyle were broken loose at its neck, it might be from an inch to an inch and three fourths from this point of the soft palate, and even much more, if it were forced up into the zygomatic fossa by the lower fragment. Therefore exploration could not determine any thing as to the injury, for the mucous membrane, soft palate, constrictor, stylo-hyoid digastric, and internal pterygoid muscles, ligaments, internal maxillary and other nerves, otic ganglion, eustachian tube, etc., are in the way. Nor can inspection of the teeth determine as to fracture in the neck of the condyle, as shown by Case 6, in which, judging by the teeth alone, the neck would appear to be fractured, whereas the injury was in the inter-articular fibro-cartilage.

It is therefore indispensable to examine carefully upon the outside of the jaw around its body, and on the ramus near the ear and zygomatic process to complete the diagnosis.

The following cases are conclusive, not only in respect to the necessity of further information of these parts being placed before surgeons, but also of the advisability of correcting Professor Hamilton's translation of Malgaigne's report on fracture of the neck of the condyle.

C. B., 24 years old, was run over by a steam fire engine Oct. 11th, 1865, and after several wounds on his scalp and chin were dressed, his jaw being fractured, he was sent to the City Hospital, Brooklyn; on arriving there, he had recovered sensibility, but was unable to swallow.

Oct, 12. The pain in left ramus and angle of the jaw being so severe, the patient could not lie on his left side, and could scarcely speak so as to be understood. The front of the jaw had gone back very much, and the surgeons endeavored to bring it into place, but could not keep it forward.

Oct. 13. With assistance of other surgeons attempts were made in various ways to hold the jaw in place, but without success. Swelling and pain undiminished.

Oct. 14. Several other surgeons were present, but on examination it was decided not to do anything until it could be discovered where the jaw was fractured, as the swelling still continued.

Oct. 15. Patient could, with difficulty, swallow water or wine in small quantities, To this time he had been fed through a small tube, by the House Surgeon.

Oct. 16. Swelling and pain abated somewhat, and patient discovered two of his teeth out of place, and called the House Surgeons' attention to their condition, who said they had not discovered *that* before.

Oct. 17. Patient's condition improved but attempts to bring the jaw into place were still unsuccessful. The chief surgeon remarked that the jaw was broken in three places.

Oct. 18. Patient went to the College of Physicians and Surgeons, New York, and in clinic was advised to get his teeth wired by a Dentist, and that, in nine cases out of ten, it was unnecessary to set the jaw at all.

Oct. 19. At the City Hospital, Brooklyn, nothing was done, and the patient left for home, where he was attended by the chief surgeon and assistant, who attempted to hold the jaw in place by gutta-percha moulded to the upper and lower teeth, assisted by a bandage supporting the chin and fastened over the head. This, like all previous attempts to bring the jaw into place, was insupportably painful, and patient was requested to return to the hospital to have it tried again under chloroform.

The application of gutta-percha in the hospital was, however, unsuccessful. The patient afterward made a second visit to the College of Physicians and Surgeons, N. Y., and was told that it was only necessary to wire the teeth to prevent the jaw from going farther back. The Surgeon in Brooklyn finally as a *dernier ressort* sent him to me to be treated for three fractures. "Fracture neck of both condyles—also fracture of body of bone."

When he presented this memorandum, his chin was supported by a bandage over the head. I found the condyles of his jaw, their necks, and his ears in good condition, but the parts under the angle of each ramus were still swollen and tender. He was very muscular, his neck being unusually large; and while the swelling caused by the accident moderated, it was superadded to by the efforts of the surgeons to pull the rami forward, in order that the lower front teeth should come to their place against the upper ones; from which they were now over $\frac{3}{8}$ of an inch back. But if the falling back had arisen from injury in or near the articulation of the condyles in the glenoid fossæ, it could result only from the condyles being forced into the ears after crushing the auditory process, which gives form to the front of the meatus; or by fracture in the neck of the condyles. Each condyle, however, as before intimated, was in its right place in front of the ear, and both came forward without pain to open the mouth; the movement being felt distinctly by the finger back in the ear and in front of the tragus. The back border of each ramus could be followed down from its condyle to its angle; while in the mouth the front borders were found perfect from the outerside of the back teeth up to, and including, each coronoid process.

In fact there was but one fracture, that which loosened the two teeth as discovered by the patient on the fifth day after the injury.

This break through the socket of the canine tooth, so near the centre of the arch of the jaw, allowed his swollen muscular neck to push the angles of the jaw out, thus as the imposts of the arch of the jaw spread apart, its crown, the chin, fell back, and this receding was increased by the muscular action brought to bear on the chin in controlling the head. The jaw held at the time of the accident fifteen teeth, all except the left molar, and, although the right canine and bicuspid were lost by the fracture, there still remained four strong teeth in the right fragment.

A stiff rubber splint was applied on November 5th, which inclosed all the teeth of both fragments, the angles being drawn in so as to bring the front teeth forward. The splint held the parts in place, by its fit on the teeth, without any other fastening, it being on the same principle as that applied to the patient in the Bellevue Hospital in January 1864, described in Case 4.

The food was masticated between the top of the splint and the upper teeth, throughout the treatment, as the jaw was allowed its natural movements, no bandage or anything external to the mouth being used, the patient attending to his business as before the accident. The bone united well.

The result confirmed my diagnosis that there was only one fracture—that in the body of the jaw, and that there was no injury whatever in or near the condyles.

Early in November, 1865, I was called upon by the medical attendant of a patient 62 years old, who had, in getting out of a stage, fallen on the curbstone, and received on the chin, a cut an inch and a half long. After this was dressed, a well known surgeon was called in, who, on examination, said the jaw was fractured through the neck of each condyle, and applied a bandage to hold it against the upper teeth. The patient was enjoined not to talk or open his jaw even to eat, his communications to be made in writing. The bandage soon caused great suffering, and turned the parts beneath it purple, so much so, that various contrivances, such as pads of lint, cork, and pasteboard were resorted to, but proved unserviceable, while the bandage required constant attention.

After hearing the Doctor's description, I felt assured that the diagnosis was incorrect. The accident occurred October 23rd, and the bandage had been applied at once, yet during about two weeks, while much suffering had resulted, no bad symptoms had been manifested in the ears, nor in the brain, although the man had struck on his chin. Even at sixty years of age, a blow on the side of the face might break the neck of the condyle, but I was not prepared to believe that the necks of both condyles had given way, through a blow directly on the front of the chin, without injury to the meatus of the ear, or to the glenoid cavity, or some other part of the temporal bone. My visitor, however, seemed certain that his eminent surgical colleague could not be mistaken. A second interview, some days after, made no special change in our respective opinions.

The patient's friends, however, felt so much concerned, that they were determined to have me called in consultation. The result was that I met the gentlemen having charge of the case at the patient's house on Nov. 16th, 1865.

In talking over the injury, before seeing the patient, the surgeon told me that the necks of the condyles were broken.

I asked as to their position; he replied that they were in their right places; that they had been drawn forward and inward, but that he had set them with his finger from the inside of the mouth. I told him he must be mistaken, as the condyle could not be set in that way. He said it was so given in the books.

We then saw the patient, who handed me a slate, on which he had written, among much other information, that before the accident his lower front teeth on one side had projected beyond the upper, while on the other side they were even.

I found his head and jaw surrounded by a bandage sixty feet long, beneath which on each side a splint of lint and plaster extended from the ear down over the ramus under and outside the body of the jaw to within half an inch of the corner of the mouth. These splints had been on four days. For the first twenty days he had worn ninety feet of bandage, but as the crepitation continued, it was thought necessary to apply the splints, and thirty feet of the bandage was left off. The patient's jaw was over to the left of its usual place, and he held it firmly closed, as if he feared it would fall off. The left lower front teeth were an eighth of an inch back of the upper, while the cutting edges of the lower right lateral incisor and canine teeth, with the front of the latter slanting to nothing at the gum were broken off.

This was done in the fall, as the chin struck so as to drive the jaw back, and the inside of these teeth struck against the outside of the upper ones.

The bandage and splints being removed, I passed my finger up the front of each ramus, and found them all right; then around the body, and found it unfractured. I then examined the necks of the condyles externally, and they with the parts around were in good condition. I told the patient to open his mouth; this he did without any pain, and he bit at my finger in front, and on the right, and the left naturally, the forward and lateral movements being unrestricted. The crepitation continued as heretofore, but it did not arise from any part of the bone, there being no fracture whatever. On the back border of the left ramus there was a very painful swelling about $\frac{5}{8}$ of an inch above the angle, extending up about three quarters of an inch; this perhaps arose at the time of the fall from the sudden strain of the back fibres of the masseter muscle. The condition of the muscles was now such as prevented them from holding the jaw forward in its former position. Its usual position, however, was not normal, its condyles having been held a little forward in the glenoid cavities to suit the irregular position of the teeth. Now with the muscles irritated by the bandage and splints, and held under unnatural strain by the fears of the patient that the condyles would get out of place, and this dread kept up by the noise made by the muscles and ligaments in their strained and unnatural condition being wrongly attributed to the friction of the fractured surfaces of the bone, it was hardly possible for the jaw to come into place, especially as the points of the teeth which had controlled the jaw were now broken off.

I advised that he wear the bandage slack until he acquired confidence; told him to use his jaw when eating, to walk and ride out when he felt disposed, and to talk as much as he pleased. On the second night his bandage getting out of place as usual, he slept without it. In the morning, November 18th, he went down town to his office without any thing on his jaw.

It grew rapidly strong, and came into place without anything further being done to it, except that one or two teeth were rounded off a little with a file.

The left ramus above the angle was tender to the touch for several months.

On November 19th, these two men, each treated for fractures through the necks of both condyles for more than three weeks, were in my office, rejoicing over their deliverance.

The only fracture which ever existed of the five they had been treated for, that in the socket of C. B's. right canine tooth, was even then so united as to leave no apprehension, the splint being removed at pleasure.

In the first case the one fracture misled the surgeons; in the second case the blow on the front of the jaw was stated to have caused an injury which at the best is very severe, and sometimes irreparable. Thus these patients were made miserable, and their sufferings very much aggravated, by those who were called in to cure them.

The importance of all this detail is now seen; it is not merely to show how the very few cases of injury in the superior terminations of the ramus of the lower jaw are to be diagnosed and treated; but also to open the way for the very much more serviceable qualification of being able to decide intelligently between very trifling injuries, and those of grave character; that is to make a correct diagnosis.

Now of the score of practitioners whose efforts caused so much anxiety and suffering to those two patients, many were of the highest standing and the results of their treatment demonstrate the propriety of the publication in 1867 of my views on the "Muscles Concerned in the Movements of the Lower Jaw." Of course, ignorance in respect to the action of these muscles was not confined to the surgeons of New York and Brooklyn; nor to those in Philadelphia who were connected with the University of Pennsylvania, for in Professor S. D. Gross' System of Surgery, Vol. 1, page 894, published in 1866, we read: "A fracture of the *neck* of the bone is easily detected, unless the subject is very fat, by the crepitation produced on moving the jaw, by the preternatural mobility in front of the ear, and by the manner in which the body of the bone is dragged forward by the action of the external pterygoid muscle. Similar symptoms will characterize fracture of the condyle of the bone."

Here this muscle which opens the mouth by pulling the condyle forward, and thus throwing the body of the bone down, is said to drag this last mentioned part of the jaw forward after it, the muscle is, by fracture of the neck of the condyle, separated from the body of the bone.

Thus with the surgeons mistaken as to the muscles which open their own mouths, they necessarily blunder in many cases far simpler than these now presented ; and nearly all who, at this period, write with the professed object of instructing them show no better knowledge of the muscles involved than those of twenty years ago.

*To be Continued.*

